# Hendra Virus Infection Dynamics in the Grey-Headed Flying Fox (*Pteropus poliocephalus*) at the Southern-Most Extent of Its Range: Further Evidence This Species Does Not Readily Transmit the Virus to Horses

**DOI:** 10.1371/journal.pone.0155252

**Published:** 2016-06-15

**Authors:** A. L. Burroughs, P. A. Durr, V. Boyd, K. Graham, J. R. White, S. Todd, J. Barr, I. Smith, G. Baverstock, J. Meers, G. Crameri, L-F Wang

**Affiliations:** 1 Commonwealth Scientific and Industrial Research Organisation, Australian Animal Health Laboratory, Geelong, Victoria, Australia; 2 City of Greater Geelong, Geelong, Victoria, Australia; 3 School of Veterinary Science, University of Queensland, Gatton, Queensland, Australia; 4 Program in Emerging Infectious Diseases, Duke-NUS Medical School, Singapore; Metabiota, UNITED STATES

## Abstract

Hendra virus (HeV) is an important emergent virus in Australia known to infect horses and humans in certain regions of the east coast. Whilst pteropid bats (“flying foxes”) are considered the natural reservoir of HeV, which of the four mainland species is the principal reservoir has been a source of ongoing debate, particularly as shared roosting is common. To help resolve this, we sampled a colony consisting of just one of these species, the grey-headed flying fox, (*Pteropus poliocephalus*), at the southernmost extent of its range. Using the pooled urine sampling technique at approximately weekly intervals over a two year period, we determined the prevalence of HeV and related paramyxoviruses using a novel multiplex (Luminex) platform. Whilst all the pooled urine samples were negative for HeV nucleic acid, we successfully identified four other paramyxoviruses, including Cedar virus; a henipavirus closely related to HeV. Collection of serum from individually caught bats from the colony showed that antibodies to HeV, as estimated by a serological Luminex assay, were present in between 14.6% and 44.5% of animals. The wide range of the estimate reflects uncertainties in interpreting intermediate results. Interpreting the study in the context of HeV studies from states to the north, we add support for an arising consensus that it is the black flying fox and not the grey-headed flying fox that is the principal source of HeV in spillover events to horses.

## Introduction

Bat species of the genus *Pteropus*, or “pteropid bats”, are geographically distributed throughout the tropics and subtropics of Asia including the Indian subcontinent, Australia, East Africa, and a number of remote islands in both the Indian and Pacific Oceans [1]. At least 63 extant species belong to this genus [2]. Pteropid bats are important reservoirs of zoonotic viruses that cause severe disease and death in humans in many parts of the world [3]. The most pertinent example is the association of pteropid bats with the cross-species transmission of two henipaviruses: *viz*. Nipah virus (NiV) in southeast and southern Asia, and Hendra virus (HeV) in Australia [4,5].

Cross-species transmission or ‘spillover’ of HeV has resulted in severe disease and death in over 70 horses in Australia [6]. Zoonotic transmission of HeV from infected horses to humans has resulted in seven human infections with four consequent deaths [7]. Serological evidence of infection with HeV has been shown for all four species of pteropid bat that occur in Australia, throughout their respective distributions [8,9]. However, the cases of HeV disease in horses and humans have only occurred in tropical and subtropical regions of the country [10,11]. By contrast, no cases of HeV infection in horses or humans have been reported in the southern, temperate regions of Australia despite opportunistic and targeted testing of horses since 1995 [12]. These findings suggest a difference in the epidemiology of HeV transmission from pteropid bats to horses between northern and southern locations.

There is evidence to suggest that certain species, namely the black flying fox (BFF, *Pteropus alecto*) and the spectacled flying fox (SFF, *P*. *conspicillatus*) play a more important role in the spillover of virus to horses compared to the grey-headed flying fox (GHFF, *P*. *poliocephalus*) and the little-red flying fox (LRFF, *P*. *scapulatus*) [13–18]. Considering the restricted tropical and subtropical distribution of the BFF and SFF, this scenario aligns with the lack of HeV detection—and disease—in horses in southern, temperate regions. The reason for one species acting as a more competent source of virus over another is unknown but may be related to the frequency and/or amount of virus shed in urine, an important vehicle for transmission [15,17,18].

Hendra virus antibody prevalence data are available from bats throughout the country [9,19–21]. Conversely, HeV pooled urinary excretion prevalence data are only available from bats in regions where HeV spillover has occurred [13,16,18,22]. In such regions, mixed-species colonies are common and make it difficult to determine species differences in HeV excretion dynamics. Difficulties arise due to the mobility of the animals and the common method of analyzing under-roost pooled urine samples to detect virus [13,22]. In these studies, colony-level viral excretion prevalence of HeV range from 0% to 67% with maximum values obtained around periods of spillover [10,13,22]. No measurements of HeV excretion prevalence exist for bats sampled in southern regions where spillover is not recorded and where single-species colonies are more common. Thus, given current available data, it cannot be determined whether the lack of HeV cases in southern locations is due to a lack of virus excretion or to differences in downstream steps of the transmission chain from bats to horses [23].

The overall objective of the current study is to provide data regarding HeV excretion prevalence for pteropid bats sampled in a southern location where no cases of HeV spillover have been identified. For this we used a sampling methodology similar to that reported from related investigations of HeV and other paramyxoviruses in pteropid bats in more northern locations [13,18,22]. This methodology allowed for a direct comparison of excretion patterns. Furthermore, the sampled colony consisted of a single species, the GHFF, which allowed a species-specific inference of reservoir competence for HeV spillover to be made.

## Materials and Methods

### Sampling

#### Animal ethics

Approval for all work involving bats was given by the Animal Ethics Committee (AEC) of the CSIRO Australian Animal Health Laboratory (AAHL) prior to commencement of this project. For colony-based urine collections, approval was given under AEC 1412, and for the trapping events under AEC 1480. Furthermore, research permits under the Wildlife Act 1975 were granted by the Victorian Department of Sustainability and Environment for all work involving bats. Urine collection permit numbers were 10005406 and 10006810. Trapping was covered under permit number 10005979.

#### Repeated cross-sectional collection of urine samples

**Fig 1:**
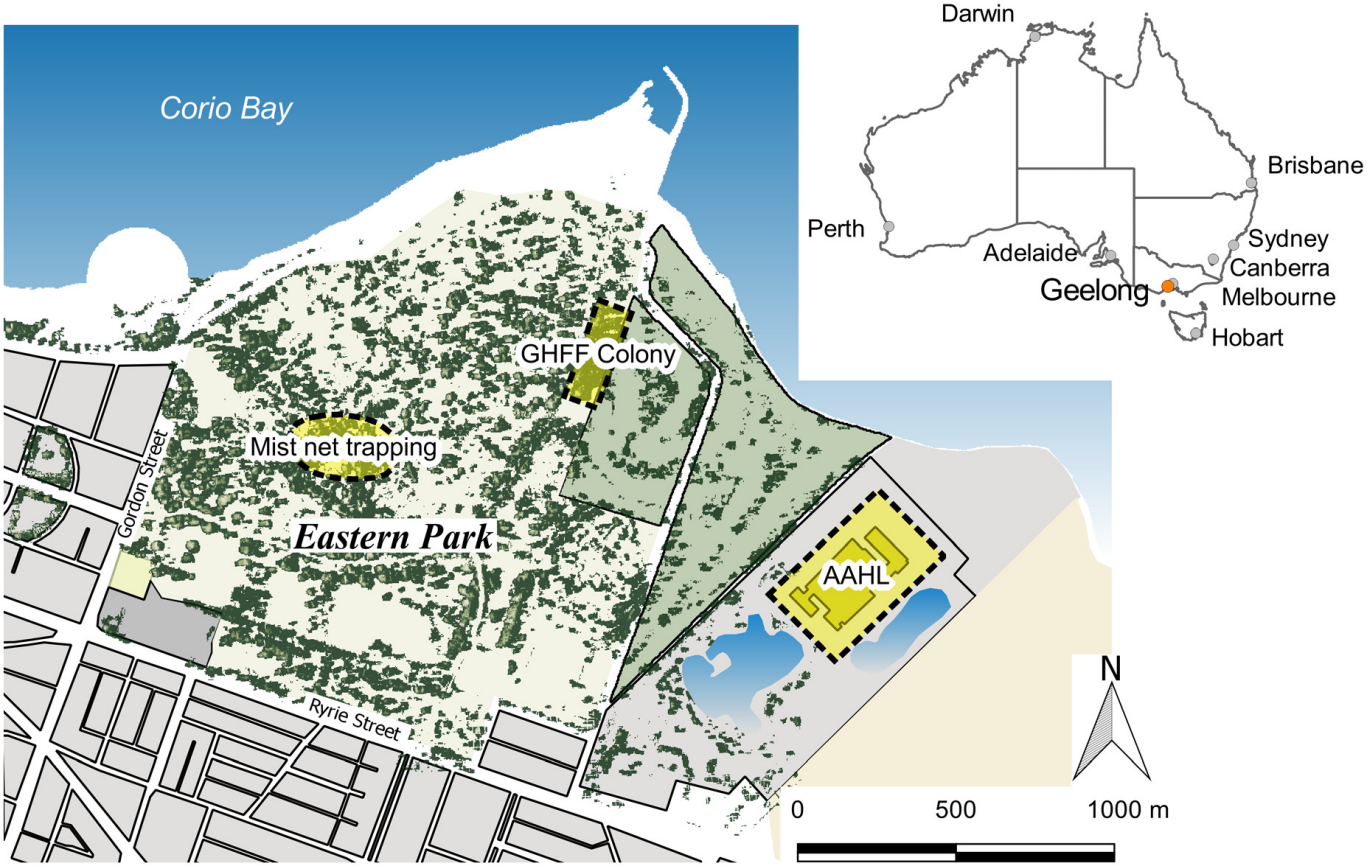
Map of key study locations. The east Geelong area, showing the relationship between the grey-headed flying fox (GHFF) colony, the Geelong Botanic Gardens where the mist net trapping was undertaken and AAHL where all the laboratory testing was conducted.

**Fig 2:**
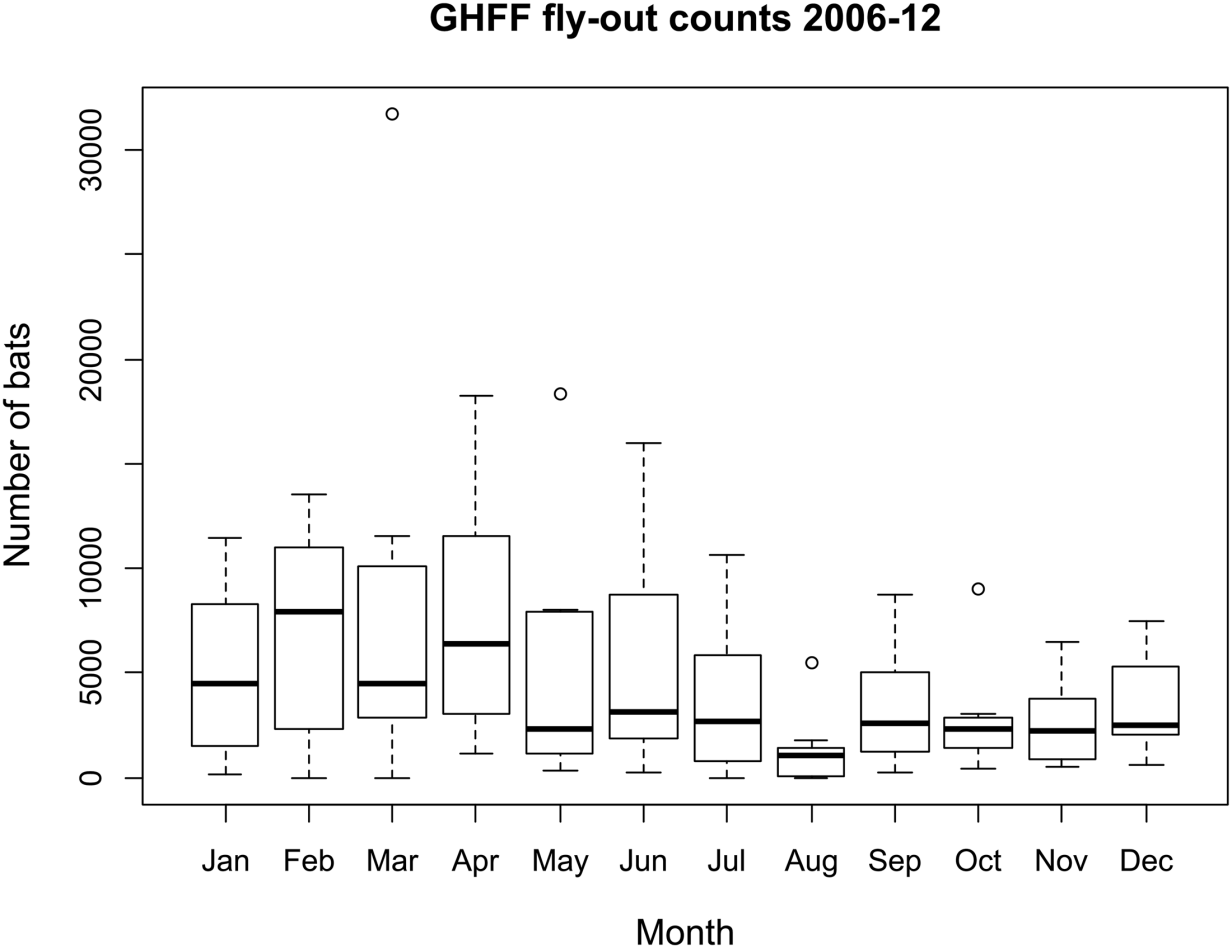
Colony size by month of observation (2006 to 2012). Box plot of the number of bats observed in the Geelong colony at fly-out by month of observation from 2006 to 2012.

The sample colony is located within the city of Geelong, Victoria, Australia [approximate colony GPS coordinates: Lat. -38.14, Long. 144.38] (Fig 1). Over the sampling period (2010–2012) and from subsequent observations, only the GHFF was observed. The colony was thus considered to be mono-specific and all indirect sampling was presumed to be from this species. The colony size was monitored on a monthly basis using fly-out counts [24,25]. Over the sampling period, the colony size varied seasonally from a minimum of approximately 800 individuals in winter/spring to a maximum of 14,000 in the summer/autumn. Monthly colony size over a seven year period is shown in Fig 2.

#### Pooled urine sampling

Pooled under-roost urine collections were performed, where possible, one morning per week from June 2010 until July 2012 –a sampling period of 109 weeks, approximately 26 months, of which 88 weeks contained a successful sampling event. Collections were conducted 2 to 5 times per month, with no months missing from the dataset. The number of pooled urine samples collected per month ranged from 20 to 50. Over 88 sampling dates, a total of 872 pooled urine samples were collected. The collection procedure followed closely that previously described [13], generally involving the placement of four plastic sheets (2.6 m x 3.6 m) each in separate locations underneath trees with roosting bats. The initial positioning of the sheets was not done at random, but rather the sheets were located preferentially under trees with a high density of bats. Locations of the sheets were kept approximately constant from week to week. Sheet placements were completed before dusk and urine collection performed the following morning, one to two hours after the dawn. If heavy rain occurred during the night of the collection urine sampling did not proceed. Personal protective equipment worn included overalls, hat, shoe covers, nitrile gloves, disposable face mask, and eye protection.

For a given sheet, neat (undiluted) urine was collected using disposable pipettes and pooled into 2 mL screw cap tubes (Sarstedt) containing 100 μL viral transport medium (VTM) (10% BSA in PBSA + 10 x Gibco Antibiotic-Antimycotic containing penicillin, streptomycin and amphotericin B). Approximately 4–15 droplets of urine were collected into each tube. A new pipette was used for each tube. For each sheet, two to three tubes of pooled urine were collected and most commonly, ten tubes of pooled urine were collected in total from all sheets at each sampling event. Care was taken not to contaminate urine samples with faeces or saliva which were also present on the sheets, although the possibility of such contamination cannot be excluded. It is important to note that it was not possible to determine how many bats contributed urine to each pooled sample. One pooled sample may contain urine from any number of bats. Furthermore, one bat may contribute urine to more than one different pooled sample. After the collection, samples were taken directly to the laboratory for storage at 4°C before processing.

#### Bat trapping

The planned schedule comprised quarterly trapping events across the year with the intention of trapping up to 30 animals per event. The Geelong colony is located in the Eastern Gardens public park in a tall stand of radiata pines of approximately 30 metres in height (Fig 1). The roost location and height made trapping within the roost impossible with available equipment and permissions. However, bats were identified socialising and feeding in the nearby Geelong Botanical Gardens during their return to the roost prior to dawn, and the location and height of animals within this site facilitated trapping. For this reason all trapping events were conducted just prior to dawn at this location [approximate trapping GPS coordinates: Lat. -38.15, Long. 144.37] (Fig 1). A 12 metre high mist net was used. In general, even when a large number of bats were present in a colony, a lower than expected number of bats were caught at each trapping event (range: 6 to 35 bats). If trapping occurred over two consecutive days, the number of bats caught on the second morning was smaller than that of the first. For this reason trapping was avoided when the colony size was small.

For early sampling events, isoflurane was used to anaesthetize bats during sample collection; however for later sampling events anaesthesia was not used, as it was found that maintenance of body temperature and recovery was better in bats that were not anaesthetized. An amendment to the animal ethics protocol was accepted by the AEC which allowed us to sample without anaesthetic agent. After sampling, bats were placed into pillowcases to recover before release. No animals were sacrificed for this project. Individual bat characteristics of sex, weight, forearm length, and age category were recorded. Weight was measured to the nearest gram and forearm length was measured to the nearest millimetre. For age, each bat was classified as either adult or juvenile. This distinction was based on external characteristics of sexual maturity, such that sexually mature bats were classified as adult. For males, sexual maturity was based on testis and penis size as well as relative body size. For females, sexual maturity was based on nipple characteristics—if small and unworn the animal was classified as juvenile. Any signs of pregnancy or previous pregnancy such as enlarged and worn nipples, palpable foetus in the abdomen or milk expression from the nipples would classify the animal as an adult. In total 137 bats were trapped over 10 trapping events.

#### Blood sample collection

Approximately 1 mL of blood was collected from the trapped bats via venipuncture from the propatagial vein into both BD Vacutainer SST Serum Separation and EDTA tubes using 23 or 25 gauge needles and 3 mL syringes. Until processing in the laboratory, EDTA blood was kept on ice and the Serum Separation samples were kept at environmental temperature. Identification of the recapture of any bat was not possible during this study but re-sampling was considered unlikely, given the size of the colony.

### Specimen processing and testing

#### RNA extraction from pooled urine samples and storage

From each pooled urine sample 500 μl was added to MagMax lysis buffer (Applied Biosystems) and the RNA was extracted using the MagMax Viral RNA Isolation Kit (Cat No. AM1836) on the MagMax Express 96 automated extraction unit (Applied Biosystems). After extraction, RNA was stored at -80°C.

#### Plasma and serum extraction from blood samples and storage

Blood samples collected in EDTA were spun at 1,000 g for 10 minutes. Plasma was then collected and stored at 0034°C. Blood samples collected into Serum Separation tubes were allowed to clot for a minimum of 30 minutes at room temperature before centrifuging at 2,000 g for 10 minutes at 25°C. Serum was collected and stored at -80°C. Before serological analyses, plasma and serum samples were first heat treated at 56°C for 30 minutes to inactivate complement.

#### Multiplex Luminex nucleic acid detection assay

For an efficient and reliable assessment of the longitudinal excretion of multiple paramyxoviruses, a multiplex Luminex nucleic acid assay was developed by our laboratory and was chosen as the screening tool [26]. Analytical sensitivities of these assays have shown to be on par or even to exceed, that of quantitative PCR assays designed to detect henipaviruses [26,27]. The assay used in this study follows the methodology of bat virus panel assay 1 (BVPA-1) as previously described by Boyd et al. [26]. The panel assay used in this study contains the same viral targets as BVPA-1 except for Nipah virus Bangladesh/Malaysia (NiV-BD/NiV-MY) and Tioman virus (TioV) which were not included. Thus the assay used was designed for the specific and simultaneous detection in pooled urine of the following bat paramyxoviruses: HeV, Cedar virus (CedPV) [21], Yeppoon virus (YepPV) [22], Grove virus (GroPV) [22], Menangle virus (MenPV) [28], Hervey virus (HerPV) [22], Teviot virus (TevPV) [29], Yarra Bend Paramyxovirus (YarPV) [26], and Geelong Paramyxovirus (GeePV) [26].

The details of the first round amplification, target-specific primer extension and the microsphere hybridisation are previously described in Boyd et al. [26]. Briefly, a one-step RT-PCR was performed using the Superscript III One-Step with Platinum Taq kit (Invitrogen). Unincorporated dNTPs and primers were removed by treating with ExoSAP-IT (Affymetrix). A target-specific primer extension (TSPE) reaction followed to incorporate the TAG sequence and biotin-labelled cytosine into the PCR products. The PCR products then underwent hybridisation to the MagPlex-TAG microspheres. Following conjugation with streptavidin-R-phycoerythrin (Invitrogen), products were assayed using the Bio-Plex Array System integrated with Bio-Plex Manager software (v 6.0) (Bio-Rad Laboratories, Inc., CA, USA). Samples were analysed at a high reporter target channel (RP1) setting with 100 beads of each bead set analysed per well. Fluorescence was measured as units of Median Fluorescence Intensity (MFI). A positive result was defined as an MFI greater than three times the average MFI of known negative controls [27,30].

#### Detection of HeV and CedPV antibodies using a Luminex assay

Antibodies against HeV and CedPV were determined using an indirect binding Luminex assay described previously by Bossart et al. [31]. Briefly, HeV or CedPV soluble G (sG) proteins were coupled to individual microsphere sets. In both assays a predetermined number of polystyrene or magnetic beads (Fisher Biotec Pty Ltd, Australia) were added to each well and then mixed with test sera at a dilution of 1:50 per sample. Bound antibody was detected using biotinylated Protein A (Pierce, Rockford, USA) together with biotinylated Protein G (Pierce, Rockford, USA) followed by streptavidin–phycoerythrin (Qiagen Pty Ltd, Australia). Results were recorded as Median Florescence Intensity (MFI).

**Fig 3:**
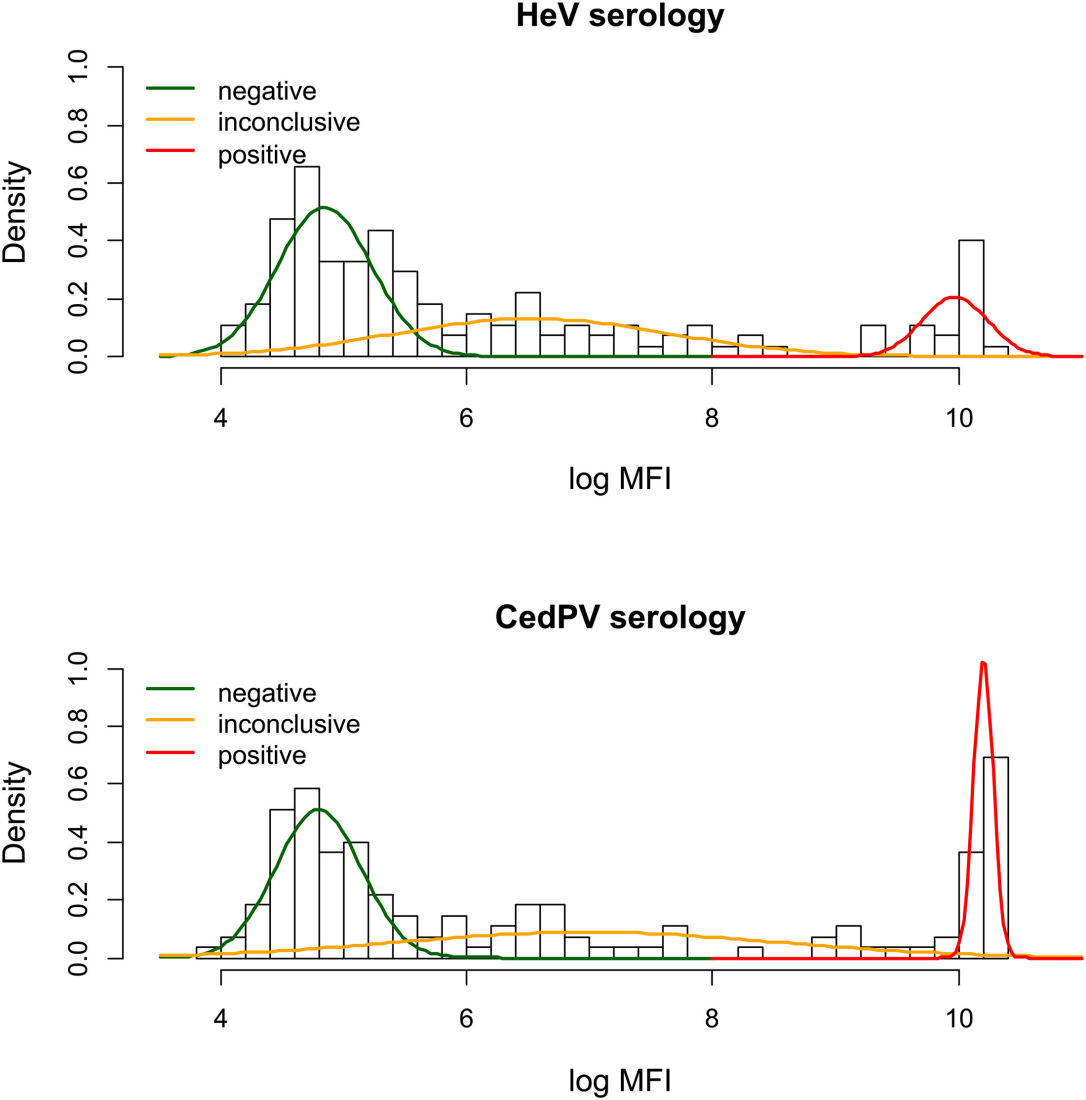
Density histogram and overlaid mixture model plots for the natural log MFI of HeV and CedPV serological responses. Upper and lower thresholds were determined to be the intersection points of the three curves and then back-transformed to give the final MFI cut-off point. Thus the lower threshold for the HeV serology was determined as the natural antilog of 5.56 (i.e. e^5.56^ = 259.7).

Thresholds for the Luminex serological assay have not yet been definitively assigned to Australian fruit bats, owing to the lack of negative and single infection control serum. To overcome this, we used a finite mixture model approach as outlined in Boyd et al [26]. In brief, the approach aims to identify different populations on the basis of the distribution of the assay parameter, which in our case was the MFI. Following the modelling, thresholds are assigned where the curves of the differing populations coincide. Exploratory analysis of the data indicated that three populations were identifiable: sero-negative, sero-positive and intermediate (Fig 3). The latter was interpreted as either the immunological responses of animals recently infected or those with waning immunity or else cross-reactions. To fit the models we used log MFI values and the function *normalmixEM* from the *R* package *mixtools* [32]. Upper and lower thresholds were estimated by back-transformation of the MFI value where the modelled distribution curves crossed.

### Data analysis

#### Estimating proportion seropositive

Sero-prevalence estimates (i.e. positives / sampled animals) were calculated for HeV and CedPV using both the upper and lower thresholds determined from the mixture modelling analysis of the log MFI curves (Fig 3). Confidence limits (95%) for these four sero-prevalence estimates were calculated using the “*epi*.*conf*” function of the *R* package “*epiR*”, using the “fleiss” method and with an initial design effect of 1. To allow for the clustered sampling, the intra-class correlation co-efficient was then estimated using the *ICCest* function of the *ICC* package, and the 95% confidence limits re-calculated using estimated design effects of 1.96 for HeV and 2.00 for CedPV.

The extent of co-seropositivity for the two viruses for each of the sampled bats was assessed using Fisher’s exact test to account for the low cell count for the doubly positive animals. The test was run using the *R* function *fisher*.*test* on both the 3x3 table with the inconclusives (n = 137) and the 2x2 table without them (n = 71).

#### Risk factor analysis of serological responses

To assess the effect of various observations and measurements taken on the trapped bats affecting the likelihood of them being seropositive to HeV or CedPV, we undertook univariate (chi square and t-tests) and logistic regression analyses. Explanatory covariates were those observed or measured during the trapping, *viz*. gender (male / female), age class (juvenile / adult), live body weight (grams), forearm length (mm) and the weight:forearm ratio. For the logistic regression analysis we undertook a standard fixed effects model on a subset of the explanatory variables (age class, forearm length and the weight:forearm ratio) in order to remove collinearity, which was assessed using the variance inflation factor (VIF). Selection for the variables was by a backwards stepwise algorithm using Akaike’s information critieria (AIC). The fixed effects modelling was then followed by mixed effects logistic regression modelling, treating the sampling date as a random effect, and thus controlling for the clustered sampling. For this mixed effects modelling, only the set of significant variables that was selected in the final fixed effects model was used.

All the risk factor modelling was undertaken within the *R* framework (version *3*.*2*.*2*), using the functions “*t*.*test*”, “*chisq*.*test*” and “*glm*” from the *stats* package, and “*vif*” and “*glmer*” from the *car* and *lmer4* packages respectively.

#### Mapping of HeV cases in relation to the distribution of pteropid bats

To place our sampling results in the wider epidemiological and ecological context, we produced maps of each of the HeV outbreaks in horses overlaid onto the distributions along the east coast of the GHFF. This mapping was also undertaken for the BFF, as the GHFF has been observed to share roosts in part of its northern range [33]. The HeV outbreak data were compiled from multiple sources, including the Queensland and New South Wales’ Departments of Primary Industries’ websites, online newspaper reports, Promed etc. Latitudes and longitudes for the towns or suburbs in which the outbreaks occurred were assigned using the *GeoNames* database (http://www.geonames.org/).

For the distribution of the flying fox species, we used habitat suitability modelling for the presence of either individual bats and/or or their roosts from 1990 to 2015 as implemented by the *Maxent* application (version 3.3) [34]. The individual data—from both sightings and museum collections—were obtained from that stored in the *Atlas of Living Australia* database (http://www.ala.org.au/). This was supplemented by the locations of the presence of the roosts, the data for which have been collected systematically since 2013 by the National Flying Fox Monitoring Program (http://www.environment.gov.au/biodiversity/threatened/species/flying-fox-monitoring).

Predictor variables used were all the BioClim bioclimatic variables, Australian Land Use and Management Classification Version 7 and the NVIS Major Vegetation Subgroups (Version 4.1). All modelling was undertaken at the resolution of 0.008 degrees (30sec) using the WGS84 datum.

## Results

### Luminex detection of Hendra virus RNA

**Fig 4:**
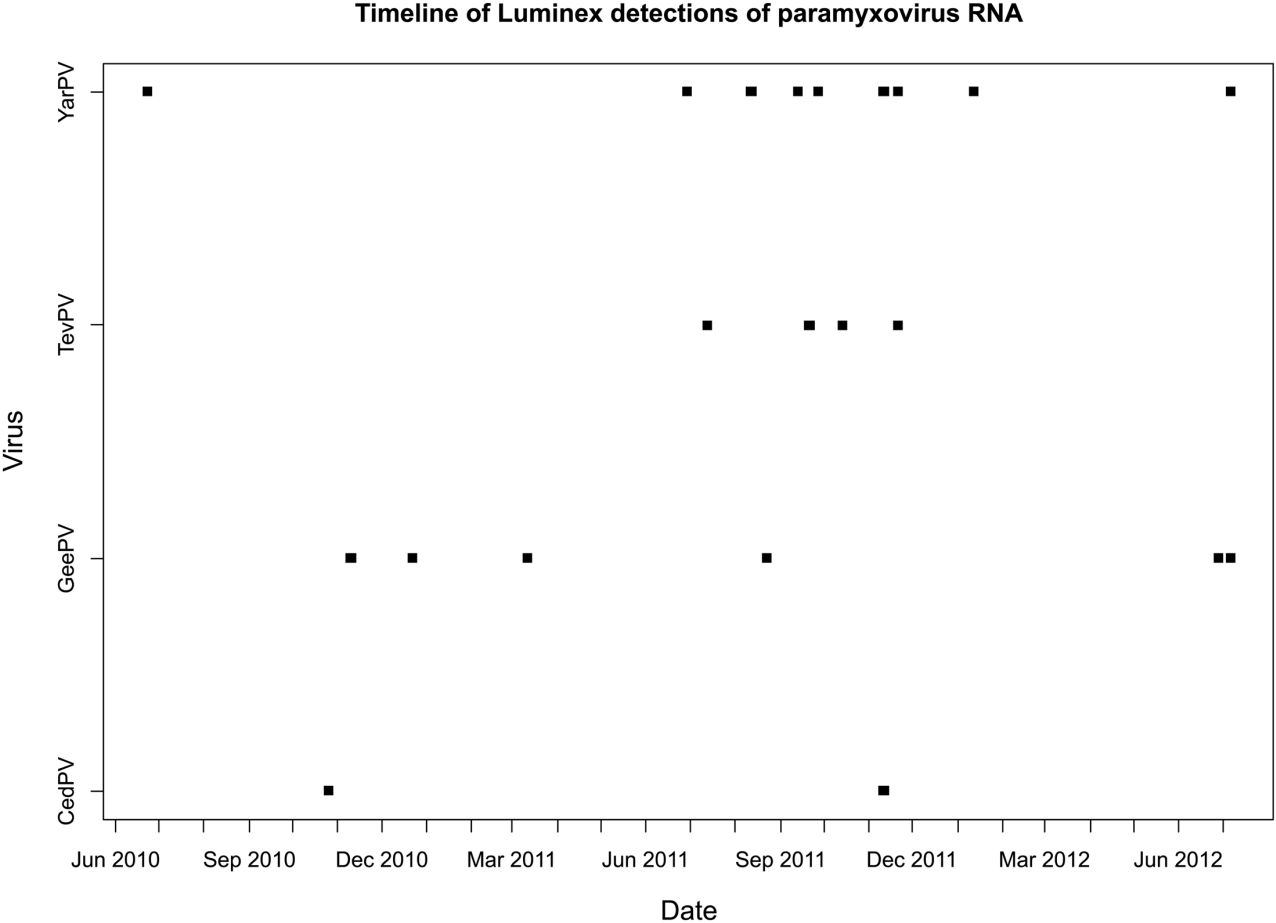
Number, type, and month of detection of non-HeV paramyxovirus sequences. Timeline of the dates when the non-HeV paramyxoviruses were detected in the urine sampling of the GHFF colony. Virus abbreviations: CedPV = Cedar paramyxovirus; GeePV = Geelong paramyxovirus; TevPV = Teviot paramyxovirus; YarPV = Yarra Bend paramyxovirus. Note that for some of the sampled dates, more than one detection of a given virus was made.

Across the entire 88 sampling events over a 26-month period, none of the 872 pooled urine samples collected and analysed yielded a positive detection of HeV RNA. Conversely, 18/88 (20.4%) of sampling events and 29/872 (3.3%) pooled urine samples respectively yielded at least one positive detection of a non-Hendra bat paramyxovirus target. Positive detections (number of detections in brackets) were made for YarPV (17), GeePV (7), TevPV (4), and CedPV (2) (Fig 4).

### Serological responses to HeV and CedPV

Line-listing of individual caught bat data including serological results and bat characteristics are given as Supporting Information (S1 Table). Applying the mixture modelling to the MFI for detection of antibodies to HeV gave a lower cut-off value of 259.7 and an upper cut-off value of 10,573.4 (Fig 3). For CedPV the comparable cut-off values were 250.6 and 21,381.6.

Applying these thresholds enabled calculations of the sero-prevalence for both of the viruses. As two thresholds are possible depending how the intermediate group are classified, then two estimates of the sero-prevalence are obtained (Table 1). Applying the lower threshold provides more sensitive estimates (i.e. all possible positives are detected) while applying the upper threshold gives more specific estimates (i.e. all possible negatives are correctly classified). For HeV the more sensitive estimate was 44.5% and the more specific estimate was 14.6%. For CedPV, these estimates were 51.1% and 21.2% respectively. As expected, allowing for the clustering of the sampling resulted in wider 95% confidence intervals for each of the sero-prevalence estimates (Table 1).

**Table 1:** Sero-prevalence estimates for HeV & CedPV using lower and upper MFI cut-offs and adjusted confidence intervals. Sero-prevalence estimates (with 95% confidence limits) for HeV and CedPV for the trapped GHFF (n = 137) using the low and high thresholds shown in Fig 3. An adjustment of the confidence limits to account for the clustered sampling is also provided.

	HeV sero-prevalence		CedPV sero-prevalence	
	Lower threshold	Upper threshold	Lower threshold	Upper threshold
**Sero-prevalence**	44.5%	14.6%	51.1%	21.2%
**Unadjusted confidence limits**	36.2%, 52.8%	8.7%, 20.5%	42.7%, 59.4%	14.3%, 28.0%
**Confidence limits adjusted for clustered sampling**	32.9%, 56.1%	6.3%, 22.9%	39.3%,62.9%	11.5%, 30.8%

Table 2 presents a cross-tabulation of HeV serological results with CedPV results using the three categories: negative, intermediate, and positive. Only 2 of the bats were seropositive for both HeV and CedPV (i.e. 2.8% of the non-inconclusives), while 32 (i.e. 45%) of the non-inconclusive animals were positive to one virus but negative to the other. The Fisher’s exact test was non-significant (p = 0.33) for the 3 x 3 cross-classification table (i.e. including the inconclusives) and the 2 x 2 table excluding them (p = 0.20).

**Table 2:** Cross-tabulation of sero-status for HeV and CedPV for the trapped GHFF (n = 137). Sero-status was based on the MFI thresholds for each virus shown in Fig 3.

	Hev Positive	HeV Inconclusive	Hev Negative	TOTAL
**CedPV Positive**	2	8	19	29
**CedPV Inconclusive**	5	16	20	41
**CedPV Negative**	13	17	37	67
**TOTAL**	20	41	76	137

**Fig 5:**
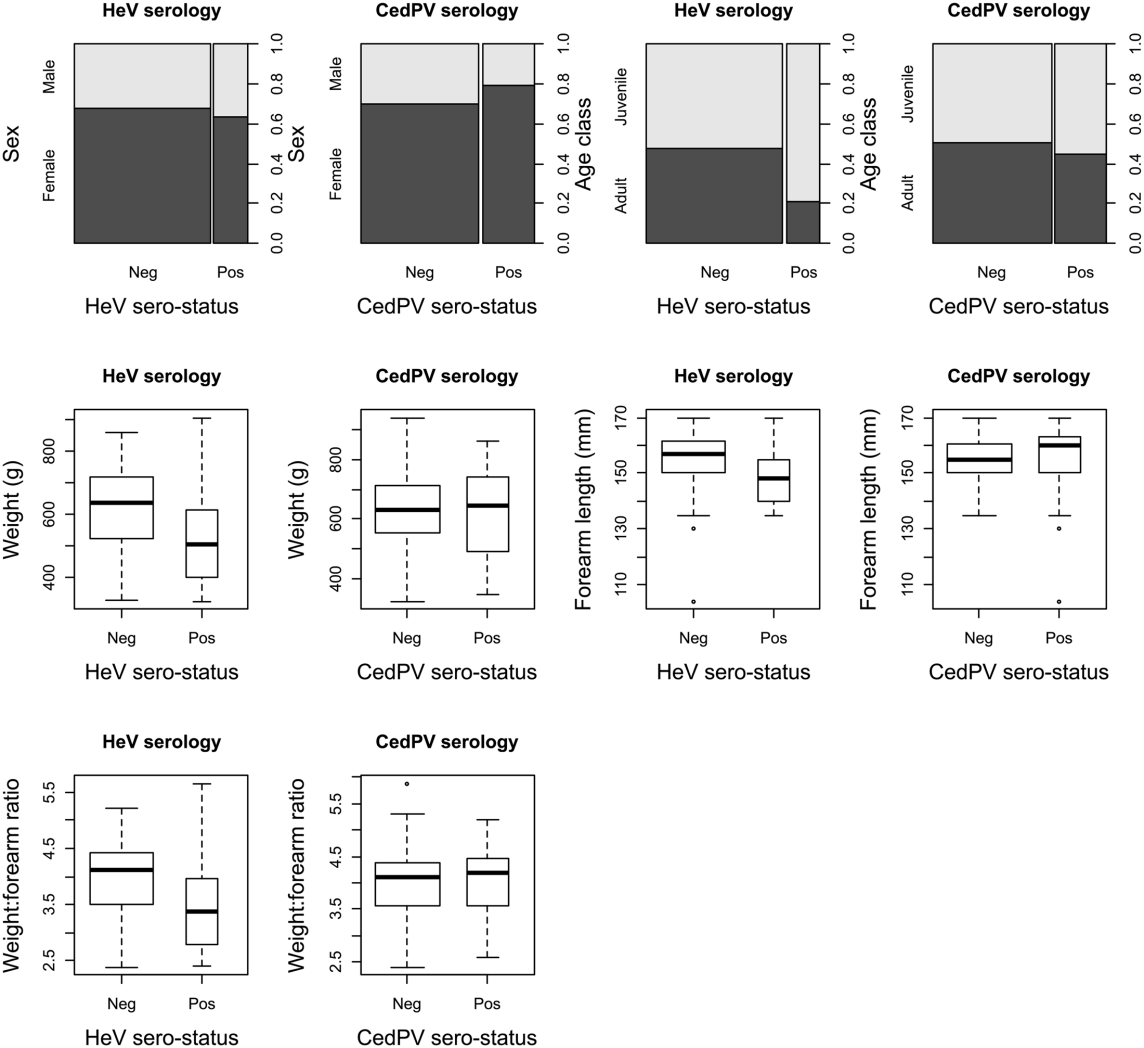
Association of bat-specific parameters with sero-status. Spineplots and boxplots of the relationships between the sero-status of the caught bats and their observed or measured parameters (sex, age-class, weight, forearm length and the weight:forearm length ratio). For both viruses, bats whose serostatus was “inconclusive” were excluded giving an analysis dataset of n = 96 for both HeV and CedPV.

The univariate risk factor analysis for seroconversion for HeV showed that the juvenile age class and a shorter forearm length were significantly associated (p < 0.05) with sero-positivity (Fig 5). The fixed effects logistic regression model showed that the juvenile age class and an interaction between this and the weight:forearm ratio to be highly significant (p < 0.01). This interaction remained significant when the random effect of sampling was accounted for (S1 Text).

For CedPV there was no significant effect of any of the observed or measured variables in either the univariate (Fig 5) or the fixed effects logistic regression analyses.

### Distribution of *P*. *alecto* and *P*. *poliocephalus* in relation to HeV outbreaks

**Fig 6:**
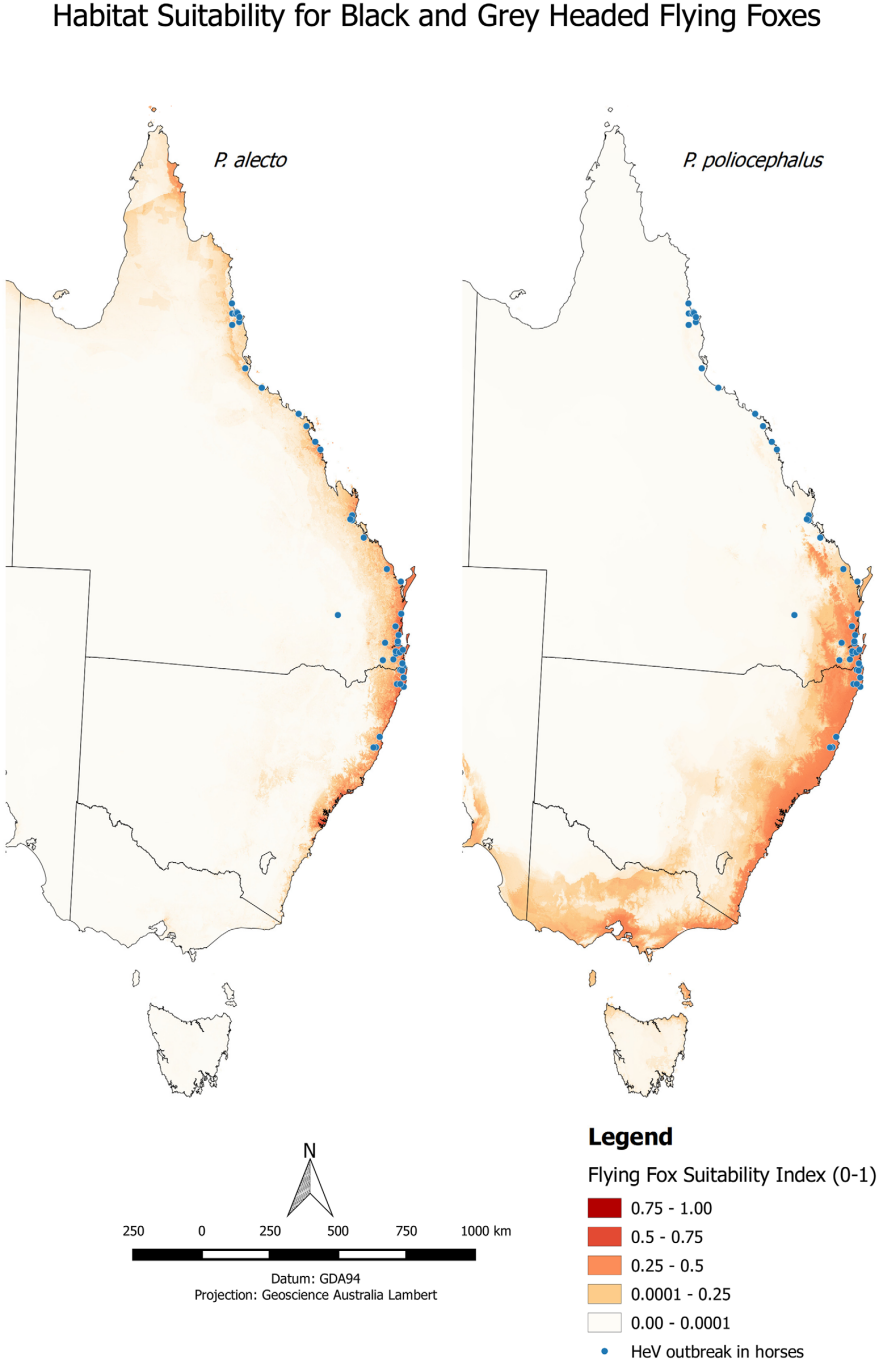
Habitat suitability models for the GHFF & BFF in relation to locations of HeV outbreaks in horses (1994–2015). Locations of the HeV outbreaks in horses between 1994 and 2015, overlaid on models for the habitat suitability of the black flying fox (*P*. *alecto*) and the grey-headed flying fox (*P*. *poliocephalus*) based on a composite of individual (1990–2015) and roost sightings (2013–15).

The habitat modelling of the two species suitability (Fig 6) broadly agrees with the analysis of the sighting records undertaken by Roberts *et al*. [33] as well as the widely used expert opinion mapping of range extent undertaken by the IUCN [35]. Important differences are that our mapping showed the expansion of the GHFF into western Victoria and South Australia, and the continued expansion of the range of the BFF down the NSW coast. With respect to HeV spillover events, none were recorded in those parts of the GFF range where there is no overlap with range of the BFF.

## Discussion

### Viral transmission dynamics

The most important finding from this study is the absence of detection of Hendra virus excretion in urine collected from the Geelong colony. The absence of detection occurred despite serological evidence of prior HeV infection in bats in the colony. Urine samples were collected frequently over multiple seasons to account for the large degree of fluctuation seen in excretion of HeV from colonies in areas where equine cases occur [13, 18]. These results lead us to conclude that the GHFFs of the Geelong colony excrete HeV rarely and/or in low concentrations. The risk of transmission of HeV from the GHFFs of the Geelong colony to horses is thus considered low. These results add support to the rising consensus that it is the BFF, and not the GHFF, which is the principal source of HeV in spillover events to horses.

Confidence in the capability of this study to detect HeV if it was excreted by roosting bats is high. The analytical sensitivity of the Luminex assay compares favourably to that of the gold standard quantitative PCR used in other studies [26]. The assay made 30 positive detections comprising four distinct non-HeV paramyxovirus sequences. Two urine samples positive for CedPV and TevPV RNA respectively were subsequently found to contain viable virus whereby it was isolated using methods specifically targeting HeV [28]. For one TevPV isolate a full genome sequence was successfully obtained [29]. This highlights the ability of the assay to detect the presence of infectious virus particles in pooled urine samples. In addition, the sampling methodology employed is similar to that successfully used by researchers in estimating HeV excretion prevalence from pteropid bat colonies in more northern locations where HeV spillover occurs [13,18,22].

Absence of detection of HeV from colony pooled urine samples is not unusual. In the investigation of HeV dynamics in pteropid colonies in Queensland and the Northern Territory over a three year period [13], of the 59 independent sampling events, 15 (25%) resulted in the detection of HeV. Of these 15 positive sampling events, most were from mixed species roosts, though all contained BFFs or SFFs. Furthermore, although GHFFs were observed during 7 of the 15 positive sampling events, these were always in association with BFFs. Of the single species roosts, virus was detected from four roosts containing only SFFs in far north Queensland and two roosts containing BFFs in southeast Queensland.

As from these tropical and sub-tropical study sites GHFF were only sampled when part of multiple species roosts, it is difficult to infer species-specific transmission dynamics from pooled urine samples. Our temperate located study is able to make such an inference as over the collection period the colony contained only GHFFs. More recent studies have examined urine samples collected from colonies of single species [16,18] and large numbers of individual bats [15,17]. The results of these four studies combined show that the rate of detection of Hendra virus by PCR is higher in BFF and SFF compared to GHFF and LRFF. When combined with the results of the studies mentioned above as well as the findings of Smith et al. [14], a consensus is emerging of the lesser role of the GHFF as a source of HeV during spillover events.

Support for this hypothesis is strengthened by the maps shown in Fig 6. The superimposition of the outbreaks onto the distributions of the GHFF and BFF clearly shows that whilst in the “hotspot” areas for HeV of southeast Queensland [23], both species are present, HeV outbreaks have occurred in areas north of the range of the GHFF. Furthermore in the areas where only the GHFF are present (southern NSW, Victoria and southeastern South Australia), no HeV outbreaks have ever been recorded. Although it was out of the scope of this study to explore in detail the epidemiology and pathobiological factors driving species differences in HeV infection dynamics, this is clearly an area warranting further research.

The results of this study may be used to better inform risk analyses for HeV spillover in non-‘endemic’ regions. The agricultural authority in Victoria believe that the highest risk for HeV incursion into the state is via movement of an infected horse rather than from local bat to horse transmission [12]. Our study adds support to this risk assessment conclusion. However the BFF is undergoing shifts in its home-range with a “latitudinal shift” southwards [33]. It would be advisable to implement surveillance of colonies with BFF incursion in order to monitor HeV excretion patterns and thus spillover risk.

### Sero-prevalence estimates

For our conclusions it was important to determine whether the ‘opportunity’ for HeV excretion from the Geelong colony existed during the study period, i.e. that bats found in the colony had evidence of prior infection. Over the last decade, the use of microsphere assays has proven an accepted and sensitive method to detect henipavirus antibody binding in fruit bat plasma and serum [36–40]. The output of these assays, MFI readings, are continuous data and present a challenge in determining meaningful cut-off values that categorise bats as seropositive or seronegative [36]. These challenges arise as the serological dynamics of infected bats (naturally or experimentally) are unknown. This makes the use of positive and negative controls for cut-off determination in these assays somewhat arbitrary. Furthermore there is no gold standard serological test to compare the results with, as even the virus neutralisation test measures different antibody binding mechanisms [36].

Our use of the mixture models to determine cut-off values follows that of Peel et al. [36] but departs in that we accept that a single cut-off is not possible for the serological profile obtained for the Geelong bats. In this way we have looked for ‘natural’ groupings of binding activity and used two cut-off values to divide these groups into negative, intermediate, and positive categories. We recognize that binding in the intermediate category may represent an important transition stage in antiviral immunity, i.e., the transition from a negative to a positive state or from a positive to negative or susceptible state. Alternatively, antibody binding in the intermediate category may represent the presence of cross-reactive antibodies generated after infection with a homologous virus. Our interpretation of field serology data has highlighted the importance of considering results on a population by population basis to generate biologically plausible groupings of antibody activity. Even using the more specific cut-off, 14.6% of bats caught from the Geelong colony showed evidence of prior infection with HeV. The lack of detection of the virus in urine is thus unlikely due to a lack of opportunity for exposure of the bats to HeV. Obtaining a narrower estimate of the time of infection would be important to better assess when and where exposure to HeV is occurring. The development of a pteropid immunoglobulin M-specific (IgM) assay for bat samples would provide better evidence of acute infection and therefore a proxy for the presence of replicating and perhaps infectious virus.

Another outcome of this study was the risk factor analysis for bat-specific factors and seropositivity. Why juveniles were more likely to be seropositive to HeV and the reasons for the complex interaction of age and weight:forearm ratio are uncertain. Plowright et al. [20] found in general that HeV seroprevalence and weight:forearm ratio shared an inverse relationship. Perhaps a distinct sub-population of bats are more susceptible to HeV infection; those that are smaller due to age or poor body condition or both. Further work is required to establish the role of age and body condition in the susceptibility to henipavirus infection. Cross-sectional serology may not be the best tool to address this question due to an inability to pinpoint the time of infection. Ideally, re-capture and sampling of the same animals over time would enable an investigation of bat-specific factors associated with seroconversion. Alternatively, once available, an IgM assay may give a better temporal assessment of the time of infection and thus factors influencing susceptibility.

It is clear from this study that there are fundamental differences in the infection dynamics of HeV and CedPV in the Geelong colony over the study period. A larger proportion of bats were seropositive to CedPV compared to HeV (21.2% vs. 14.6% using the upper threshold), no bat-specific risk factors predicted CedPV seropositivity, and CedPV was detected and subsequently isolated while no detections of HeV were made. Furthermore, there was no association between the sero-status for the two viruses, and only two bats were seropositive for both HeV and CedPV (Table 2). It is unclear whether this represents concurrent or consecutive infections [37]. Once developed, the use of an IgM assay would likely distinguish between these states in the future [37]. Just as it is problematic to assume similar virus-host transmission dynamics between host species, inferring virus-host transmission dynamics across viruses of the same genus should be avoided.

## Conclusion

The results of our study contribute to the field of emerging diseases from wildlife reservoirs by highlighting the importance of characterizing relative species competence as sources of pathogens in spillover events. Such an understanding enables more robust risk assessments and targeted preventative approaches. This study highlights that not all wildlife reservoirs are “created equal”—positive serological results from a purported reservoir are “necessary but not sufficient” to establish them as a primary reservoir in driving spillover events to humans and domestic animals.

## Supporting Information

S1 Table MFI readings of Luminex antibody binding assay using HeV & CedPV sG protein for each trapped bat including bat specific measurements. (CSV)

S1 Text R output from the generalised linear mixed effect modelling using the “glmer” function of the lme4 package for HeV. Only the 96 bats with non-inconclusive results were modelled. The 10 collection dates were treated as a random effect and age class and the weight to forearm:length ratio (“w.f”) as the fixed effects.
(DOC)
