# Supplementary material for: Hendra Virus Infection Dynamics in the Grey-Headed Flying Fox (Pteropus poliocephalus) at the Southern-Most Extent of Its Range: Further Evidence This Species Does Not Readily Transmit the Virus to Horses
Source: PLoS One. 2016 Jun 15;11(6):e0155252. doi: 10.1371/journal.pone.0155252 (PMC4909227; doi:10.1371/journal.pone.0155252)
Supplement: S1 Text — Only the 96 bats with non-inconclusive results were modelled. The 10 collection dates were treated as a random effect and age class and the weight to forearm:length ratio (“w.f”) as the fixed effects. (DOC) [file pone.0155252.s002.doc]

**S1 Text**

Generalized linear mixed model fit by maximum likelihood (Laplace Approximation) ['glmerMod']

Family: binomial ( logit )

Formula: HeV_Luminex ~ age_class * w.f + (1 | Collection_Date)

Data: HeV

AIC BIC logLik deviance df.resid

78.4 91.2 -34.2 68.4 91

Scaled residuals:

Min 1Q Median 3Q Max

-1.72030 -0.35902 -0.13113 -0.01553 2.19926

Random effects:

Groups Name Variance Std.Dev.

Collection_Date (Intercept) 2.192 1.481

Number of obs: 96, groups: Collection_Date, 10

Fixed effects:

Estimate Std. Error z value Pr(>|z|)

(Intercept) -33.435 13.880 -2.409 0.01600 *

age_classJuvenile 39.004 14.451 2.699 0.00695 **

w.f 6.419 2.814 2.281 0.02252 *

age_classJuvenile:w.f -8.541 3.072 -2.780 0.00543 **

---

Signif. codes: 0 ‘***’ 0.001 ‘**’ 0.01 ‘*’ 0.05 ‘.’ 0.1 ‘ ’ 1

Correlation of Fixed Effects:

(Intr) ag_clJ w.f

ag_clssJvnl -0.980

w.f -0.998 0.978

ag_clssJv:. 0.958 -0.994 -0.959
